# Consumption of steam explosion and fermentation-pretreated corn stover affects the growth performance of sheep by shifting the rumen microbiota community structure

**DOI:** 10.3389/fmicb.2025.1532746

**Published:** 2025-04-03

**Authors:** Yong Wang, Xuejiao Yin, Kexing Hao, Chao Wang, Wurilege Wei, Yueqin Li, Sihui Gao, Zeyu Ji, Weiheng Wang, Yuchun Xie, Changqing Li

**Affiliations:** ^1^Inner Mongolia Academy of Agricultural & Animal Husbandry Sciences, Hohhot, China; ^2^Key Laboratory of Grass-Feeding Healthy Breeding and Livestock Product Control (Co-construction by Ministry and Province), Ministry of Agriculture and Rural Affairs, Hohhot, China; ^3^College of Animal Science and Technology, Hebei Normal University of Science and Technology, Qinhuangdao, China; ^4^Key Laboratory of Specialty Animal Germplasm Resources Exploration and Innovation, Qinhuangdao, China

**Keywords:** steam explosion, corn stover, sheep, rumen microbiota, growth performance

## Abstract

Corn stover is rich in lignocellulose, which results in low digestibility. Steam explosion is a hydrothermal pretreatment widely used to improve the digestibility of plant-based materials by inducing cell wall disruption through the rapid release of pressure. However, the impact of steam explosion-treated corn stover on the growth performance of sheep consuming it remains unclear. This study aimed to evaluate the effects of steam explosion and *Lactobacillus buchneri* inoculation on the nutritional value and rumen microbiota of corn stover. Corn stover was prepared with or without steam explosion and *L. buchneri* inoculation. Eighty sheep of similar body weight (47.62 ± 0.74 kg) were allocated into two groups and fed either untreated corn stover (CON, *n* = 40) or steam explosion-pretreated corn stover cocultured with *L. buchneri* (SEC, *n* = 40). The experiment lasted 60 days, and it included 10 days of adaptation and 50 days of feeding. Our results indicated that compared with the CON group, the final weight, average daily gain, and daily dry matter intake of the SEC group were significantly reduced (*P* < 0.05). Alpha-diversity analysis showed that the Chao1 index of the rumen microbial community tended to be lower (*P* < 0.1) in the SEC group. At the genus level, the SEC group had a higher (*P* < 0.05) relative abundance of *Stylonychia*, *Paramecium*, *Treponema*, *Blepharisma*, *Neocallimastix*, *Stentor*, *Tetrahymena*, *Ichthyophthirius*, and *Pseudocohnilembus* than the CON sheep. We identified the top 15 KEGG functional terms with distinct differences between the CON and SEC groups, with endocytosis being the only pathway enriched (*P* < 0.05) in the SEC group. Based on these findings, we speculated that, compared with the CON group, the consumption of corn stover pretreated with steam explosion and fermentation may have been associated with greater energy loss via the production of more methane and lower microbial activities, contributing to the low utilization and efficiency of this feedstuff. Before the widespread application of steam explosion to corn stover, there is a need to further optimize the production process, with particular attention paid to the proportion of corn stover in the diet.

## 1 Introduction

Corn stover is one of the most common crop residues, with an annual global yield of approximately 1.15 billion tons (FAOSTAT, 2021). Although it is commonly discarded as waste, using it efficiently would be extremely beneficial for biofuel/livestock production. This would also ameliorate the land and air pollution risks currently associated with its disposal. However, the high lignocellulose content and high degree of lignification in mature corn stover hinder its efficient utilization (Wang et al., 2020). Therefore, disrupting the recalcitrant structure of corn stover and developing an economical storage method are essential steps to ensure its year-round supply and efficient use.

Steam explosion technology has emerged as a promising method for pretreating food processing byproducts because of its high efficiency, environmental friendliness, and low cost (Nie et al., 2021). Although it was originally applied in fields such as pulping and wood processing (Martin-Sampedro et al., 2014), animal feed processing (Xie et al., 2024), and bioenergy production (Hoang et al., 2023), recent studies have also suggested that steam explosion could reduce the antigenicity, β-conglycinin, and phytic acid content in soybean meal (Kong et al., 2022), and change the ruminal fermentation of corn stover based on *in vitro* findings (Wang et al., 2020). Steam explosion can degrade hemicellulose into soluble monosaccharides or oligosaccharides (Zhao et al., 2018). Under the process of Steam explosion, the content of neutral detergent fiber and hemicellulose can be reduced from 73.2 and 34.7 to 49.7 and 6.7, respectively (Shi et al., 2019). Meanwhile, the morphological structure is broken, the accessibility of cellulose is increased (2.44, 2.83, 4.08-4.33 mg/g) and the content of acid detergent lignin is decreased (17.52%) (Nie et al., 2021). This process effectively disrupts the cell walls of plant-based materials, enhancing the extraction and biological activity of their active components, which makes it a promising method for corn stover pretreatment.

However, existing research has primarily focused on the structural changes of straw induced by steam explosion technology, but the specific feed effects of steam-exploded straw remain unclear. There is also a need to further explore this technology’s acceptance by animals, its palatability, and its impact on the rumen, which is the primary site of digestion in ruminants. Specifically, although steam explosion changes the structure of lignocellulosic materials, their subsequent digestibility and utilization by animals remain unclear. This study was established to investigate the effects of consuming corn straw pretreated with steam explosion and fermentation on the growth performance of fattening sheep, such as their growth performance and serum parameters, along with rumen metagenomic sequencing results. We hope that the assessment of these factors will provide valuable insights into sustainable livestock feeding practices and promote the utilization of agricultural byproducts in animal nutrition.

## 2 Materials and methods

### 2.1 Experimental animals and diet

The animal experiment was approved by the Ethics Committee on the Use of Production Animals of the Inner Mongolia Academy of Agricultural & Animal Husbandry Science (Approval number: No.1 20231207).

This study was conducted between July 2023 and November 2023 at a sheep farm in Inner Mongolia, China. A total of 80 male Hei sheep, with a mean ± standard error (SE) body weight (BW) of 47.62 ± 0.74 kg and aged 6 months, were selected for the experiment. These sheep were randomly assigned to one of two groups with the following diets: untreated corn stover (CON, *n* = 40) and steam explosion-pretreated corn stover cocultured with *Lactobacillus buchneri* (SEC, *n* = 40). The steam explosion-pretreated corn stover was sourced from Inner Mongolia Modern Pasture Operation Technology Co., Ltd.

The sheep were kept in individual pens and provided with fresh water *ad libitum*. All sheep were fed a total mixed ration (TMR) twice daily (0700 and 1,600 h), as shown in Table 1. The experimental period was 60 days, with a 10-day period of adaptation to the experimental diets and a 50-day feeding period. The sheep were weighed on day 1 and day 60, before morning feeding, to calculate their average daily gain (ADG). Daily dry matter intake (DDMI) was determined by weighing each sheep’s daily feed supply and leftovers.

**TABLE 1:** Nutrient composition of experimental diet (dry matter basis).

Items^1^, %	Dietary treatment	
	**Corn stover**	**Steam explosion-pretreated corn stover**
**Components**		
Corn straw	26	0
Spray corn stover	0	26
Soybean meal	5	5
Cotton meal	7	7
Corn	35	35
Spouting corn husks	6	6
Corn germ meal	16	16
NaCl	0.7	0.7
Stone powder	0.6	0.6
NaHCO_3_	0.7	0.7
Premix^2^	3	3
Total	100	100
**Chemical composition**		
Dry matter	88.26	67.52
Metabolizable energy, MJ kg^1^ DM	12.10	9.47
Crude protein	14.19	15.41
Neutral detergent fiber	27.55	27.36
Acid detergent fiber	17.65	19.06
Ether extract	2.08	1.71
Ash	8.59	8.95
Calcium	0.85	0.83
Phosphorus	0.31	0.31

^1^Metabolizable energy is expressed in calculated values, while the others are measured values. DM: dry matter.

^2^The premix provided the following per kilogram of diet: vitamin A 90,000–200,000 IU, vitamin D3 30,000–80,000 IU, vitamin E ≥ 600 IU, Fe 0.3–15 mg (as ferrous sulfate monohydrate), Cu 0.2–0.6 mg (as copper sulfate), Zn 0.8–2.4 mg (as zinc sulfate monohydrate), Mn 6–100 mg (as manganese sulfate monohydrate), Se 5–10 mg (as sodium selenite), I 6–100 mg (as potassium iodide), Co 2–40 mg (as cobalt chloride), and NaCl 10%–40%.

^3^Metabolizable Energy (ME) Calculation Formula: Metabolizable Energy can be calculated using the following formula: *ME* (*MJ/kgDM*) = 0.016 × *GE* (*MJ/kgDM*) – 0.17 × *CP* (%) Where: GE is Gross Energy, in MJ/kg DM. CP is Crude Protein content, in% DM.

### 2.2 Blood and rumen sample collection

Six sheep were randomly selected from each experimental group to undergo sample collection. Blood samples (5 mL) were obtained from them on the 60th day of the feeding period, before the morning feeding, by jugular venipuncture, using coagulation-promoting tubes. The samples were then centrifuged at 3,000 × g for 20 min to extract the serum, which was subsequently divided into three aliquots and stored at −20°C for subsequent analysis.

For the rumen fluid sample collection, eight sheep from each group were randomly chosen. Approximately 50 mL of rumen fluid was collected via an oral stomach tube before the morning feeding on day 52 of the feeding period (Yin et al., 2021). To prevent cross-contamination between samples, the oral stomach tube was thoroughly cleaned with fresh warm water between each collection. All samples were immediately placed in liquid nitrogen and subsequently stored at −80°C for further analysis.

### 2.3 Serum biochemical analysis

All blood parameters were analyzed using commercial kits, following the manufacturer’s instructions (Grace Biotechnology, Suzhou, China). Serum β-hydroxybutyrate (BHBA) levels were measured using ELISA test kits (Cat. No. ZC8116602), while the absorbance (OD) was measured at 450 nm. The levels of superoxide dismutase (SOD, Cat. No. 702121), immunoglobulin A (IgA, Cat. No. 70275), immunoglobulin G (IgG, Cat. No. 70276), immunoglobulin M (IgM, Cat. No. 70277), glucose (GLU, Cat. No. 70229), total protein (TP, Cat. No. 70212), and albumin (ALB, kit 70213) were determined using commercially available kits. Serum diamine oxidase (DAO) activity was measured using a commercially available DAO kit (G0134W), and the OD was measured at 510 nm. Serum glutathione peroxidase (GSH-Px) levels were measured using a commercially available GSH-Px kit (Cat. No. G0204W), and the OD was measured at 412 nm. Serum lipopolysaccharide (LPS) was measured using a commercially available LPS kit (Cat. No. G0902W), and the OD was measured at 405 nm. Serum malondialdehyde (MDA) levels were determined using a commercially available MDA kit (Cat. No. G0109W), and the OD was measured at 532 nm and 600 nm. Serum non-esterified fatty acid (NEFA) concentrations were measured using a commercially available NEFA kit (Cat. No. G0927W48), and the OD was measured at 546 nm. Finally, serum total antioxidant capacity (T-AOC) was measured using a commercially available T-AOC kit (Cat. No. G0142W), and the OD was measured at 414 nm.

### 2.4 Metagenomic sequencing analysis

Total genomic DNA was extracted from rumen fluid samples using the PSP Spin Stool DNA Plus Kit (Invitek, Berlin, Germany), following the manufacturer’s instructions. The concentration and purity of the extracted DNA were determined using TBS-380 and NanoDrop2000, respectively. The quality of the DNA extract was checked on a 1% agarose gel.

Each DNA extract was fragmented to an average size of approximately 350 bp using Covaris M220 (Gene Company Limited, Hong Kong, China) for paired-end library construction. The paired-end library was constructed using NEXTFLEX^®^ Rapid DNA-Seq (Bioo Scientific, Austin, TX, United States). Adapters containing the full complement of sequencing primer hybridization sites were ligated to the blunt ends of the fragments. Paired-end sequencing was performed on Illumina NovaSeq (Illumina Inc., San Diego, CA, United States) at Majorbio Bio-Pharm Technology Co., Ltd. (Shanghai, China), using NovaSeq 6000 S4 Reagent Kit v1.5 (300 cycles), following the manufacturer’s instructions (see online instructions on the Illumina website: www.illumina.com).

### 2.5 Bioinformatic analysis

The data were analyzed on the free online platform Majorbio Cloud Platform.^1^ Paired-end Illumina reads were trimmed of adaptors, and low-quality reads (length < 50 bp or quality score < 20) were removed using fastp (Chen et al., 2018; version 0.20.0).^2^ Metagenomic data were assembled using MEGAHIT (Li et al., 2015; version 1.1.2),^3^ which uses succinct de Bruijn graphs. Contigs with lengths ≥ 300 bp were selected as the final assembly result, and the contigs were then used for further gene prediction and annotation.

Open reading frames (ORFs) were predicted from each assembled contig using Prodigal (version 2.6.3; Hyatt et al., 2010)/MetaGene (Noguchi et al., 2006).^4^ The predicted ORFs with length ≥ 100 bp were retrieved and translated into amino acid sequences using the NCBI translation table.^5^

Representative sequences from the non-redundant gene catalog were aligned to the NR database with an *e*-value cut-off of 1e^–5^ using Diamond (Buchfink et al., 2015; version 0.8.35)^6^ for annotation. KEGG annotation was performed using Diamond against the Kyoto Encyclopedia of Genes and Genomes database^7^ with an *e*-value cut-off of 1e^5^. Principal coordinate analysis (PCoA) was performed based on Bray–Curtis dissimilarity matrices at the genus level. To compare the general microbial functional profiles among different groups, PCoA was conducted based on the relative abundance of KEGG pathways. Alpha-diversity values were calculated using Chao1 and Shannon indices.

### 2.6 Statistical analysis

The *t*-test was used to analyze the significance of differences in growth performance, serum biochemical parameters, and rumen microbial community at the phylum level using SPSS software (version 26.0). Rumen metagenomic data were analyzed using the Majorbio Cloud Platform (Han et al., 2024; see text footnote 1). The significance of differences between groups was tested using Anosim. The alpha diversity, relative abundance of rumen genera, and KEGG pathways were compared between the two groups using the Wilcoxon rank-sum test, with a false discovery rate-adjusted *P*-value of < 0.05 being considered to reflect a significant difference (only the top 15 genera/pathways are displayed). Correlations between datasets were calculated using Spearman’s correlation coefficients.

## 3 Results

### 3.1 Growth performance and serum biochemical parameters

Table 2 shows the growth performance of the sheep. There was no difference in initial weight between the two groups (*P* > 0.05), but the SEC group exhibited significant decreases in final weight, ADG, and DDMI compared with the CON group (*P* < 0.05).

**TABLE 2:** Effects of different types of corn stover on the growth performance of sheep.

Items	Corn stover	Steam explosion- pretreated corn stover	SEM	*P*-value
Initial weight (kg)	48.58	48.66	0.7385	0.913
Final weight (kg)	61.01	57.91	1.1397	0.008
Average daily gain (g)	223.06	153.89	10.544	<0.001
Daily dry matter intake (kg)	2.10	1.31	0.089	<0.001

Table 3 shows the effects of the experimental diet on the serum parameters. The results revealed that compared to the SEC group, the CON group had higher concentrations of ALB and TP (both *P* < 0.05), but there were no marked differences in the other parameters between the treatments (*P* > 0.05).

**TABLE 3:** Effects of different types of corn stover on serum biochemical parameters of sheep.

Items	Corn stover	Steam explosion- pretreated corn stover	SEM	*P*-value
**Immunoglobulin (mg/mL)**				
Immunoglobulin A	10.10	11.08	1.645	0.565
Immunoglobulin G	18.80	18.33	2.108	0.830
Immunoglobulin M	150.00	142.50	12.340	0.558
**Metabolism**				
Glucose (mmol/L)	6.06	5.38	1.283	0.606
β-Hydroxybutyrate (μmol/L)	14.87	15.37	1.247	0.697
Non-esterified fatty acid (mmol/L)	0.51	0.45	0.214	0.780
Total protein (g/L)	131.70	122.50	4.000	0.047
Albumin (g/L)	67.80	58.75	2.453	0.005
**Antioxidant indicator**				
Superoxide dismutase	420.00	377.50	24.420	0.116
Glutathione peroxidase	150.48	149.59	2.201	0.696
Total antioxidant capacity	0.07	0.07	0.012	0.705
Malondialdehyde	0.24	0.42	0.219	0.127
**Intestinal permeability indicator**				
Lipopolysaccharide	267.88	296.39	34.854	0.434
Diamine oxidase	1.63	1.42	0537	0.696

### 3.2 Difference in rumen microbial diversity indices

Metagenomic sequencing generated a mean of 92,591,235 reads per sample. After quality control and removal of host genes, a mean of 90,824,781 reads per sample was retained. After *de novo* assembly, a total of 11,044,467 contigs were generated, with 690,279 contigs per sample.

**FIGURE 1:**
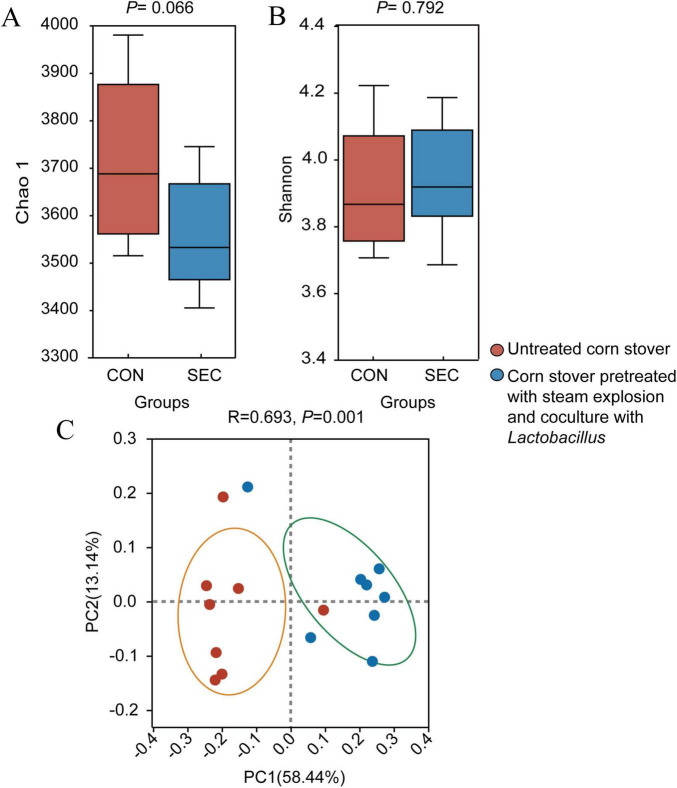
Effects of different types of corn stover on the diversity of the rumen microbial community of sheep. Estimates of species richness (Chao1; **A**) and diversity indices (Shannon; **B**) for the different groups are presented. **(C)** Principal coordinate analysis (PCoA) of the rumen microbial communities of the two groups based on Bray–Curtis dissimilarity.

Alpha-diversity analysis showed that the Chao1 index of the rumen microbial community tended to be lower (*P* < 0.1) in the SEC group (Figure 1A). Meanwhile, the Shannon index (Figure 1B) in the rumen was similar (*P* > 0.05) between the CON and SEC groups. Beta-diversity analysis and Bray–Curtis distance-based PCoA revealed that the microbial composition in the rumen was different between the two groups (Figure 1C).

### 3.3 Changes in rumen microbial composition and its correlation with growth performance

At the phylum level, the SEC group had a higher (*P* < 0.05) relative abundance of Ciliophora and a lower (*P* < 0.05) relative abundance of Firmicutes and Candidatus Saccharibacteria than the CON group (Table 4).

**TABLE 4:** Effects of different types of corn stover on the rumen microbial community of sheep at the phylum level (relative abundance > 1%).

Items	Corn stover	Steam explosion- pretreated corn stover	SEM	*P*-value
Firmicutes	47.89	25.31	4.820	<0.001
Bacteroidota	17.32	11.80	3.618	0.149
Actinobacteria	2.43	1.08	0.675	0.065
Ciliophora	0.51	1.84	0.268	<0.001
Proteobacteria	1.28	0.87	0.256	0.131
Spirochetes	0.69	1.06	0.177	0.054
Euryarchaeota	0.72	0.62	0.233	0.662
Candidatus Saccharibacteria	1.18	0.08	0.246	0.003

**FIGURE 2:**
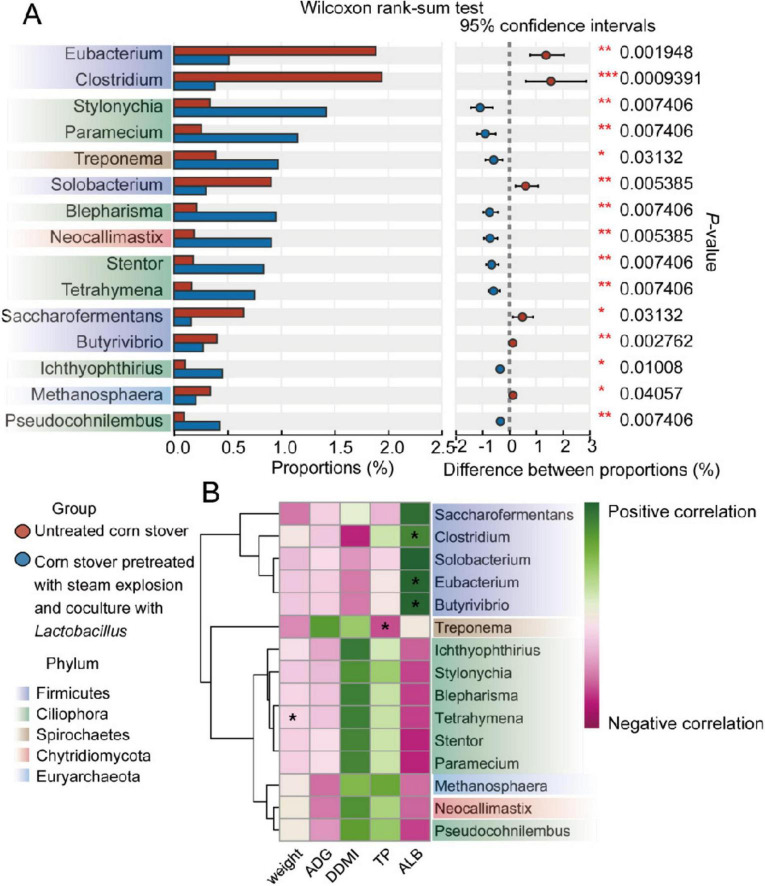
Comparison of rumen taxa identified by metagenomic analysis between sheep consuming different corn stovers. **(A)** The 15 most significantly different genera as identified by the Wilcoxon rank-sum test are presented. **(B)** The heatmap shows the Spearman correlations between biomarkers (identified by the Wilcoxon rank-sum test between two groups) and growth performance (significantly affected by treatment). *P < 0.05.

Microbial genera were also compared between the rumen microbiomes of the two groups (Figure 2A). At the genus level, the SEC group had a higher (*P* < 0.05) relative abundances of *Stylonychia*, *Paramecium*, *Treponema*, *Blepharisma*, *Neocallimastix*, *Stentor*, *Tetrahymena*, *Ichthyophthirius*, and *Pseudocohnilembus* than the CON sheep. Meanwhile, compared with the levels in the CON group, the relative abundance of *Eubacterium*, *Clostridium*, *Solobacterium*, *Saccharofermentans*, *Butyrivibrio*, and *Methanosphaera* were reduced (*P* < 0.05) in the SEC group.

To investigate the effect of the different genera abundance in the rumen on host growth, an analysis of correlations between biomarkers exhibiting significant differences between groups (TP, ALB, FW, and ADG) and microbial biomarkers was conducted, as illustrated in Figure 2B. The results showed that the final weight of the experimental sheep was negatively (*P* < 0.05) correlated with the relative abundance of *Tetrahymena*. Meanwhile, the concentration of serum TP was negatively (*P* < 0.05) correlated with the relative abundance of *Treponema.* Finally, the level of serum ALB was positively (*P* < 0.05) correlated with the relative abundance of *Clostridium*, *Eubacterium*, and *Butyrivibrio*.

### 3.4 Profiling of the functional capacity of the rumen microbiota based on metagenomic sequencing

**FIGURE 3:**
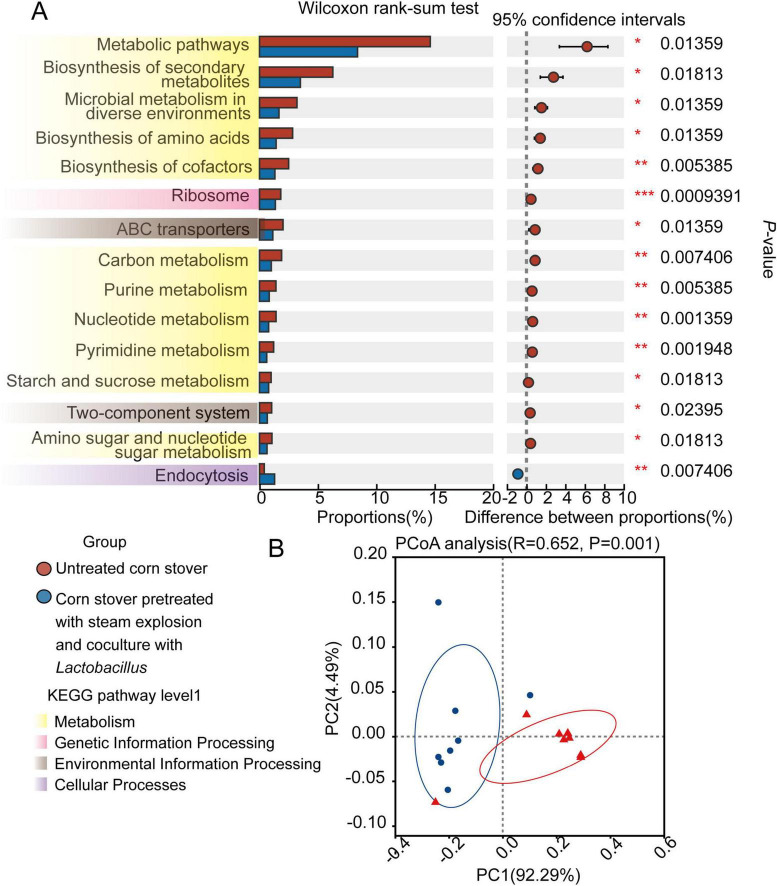
Effects of different types of corn stover on the functional capacity of the rumen microbial community of sheep. **(A)** The 15 most significantly different KEGG pathways associated with different types of corn stover, as identified by the Wilcoxon rank-sum test, are presented. **(B)** Principal coordinate analysis (PCoA) of the KEGG pathways of the two groups based on Bray–Curtis dissimilarity.

We further investigated the KEGG pathways associated with the microbiota in the rumen of sheep upon their consumption of the pretreated or untreated corn stover. In Figure 3, we present the top 15 KEGG functional terms (KEGG pathway level 3), showing differences in enrichment between the CON and SEC groups. Only endocytosis was enriched (*P* < 0.05) in the SEC group, while the other 14 pathways (metabolic pathways, biosynthesis of secondary metabolites, microbial metabolism in diverse environments, biosynthesis of cofactors, ribosome, ABC transporters, carbon metabolism, purine metabolism, nucleotide metabolism, pyrimidine metabolism, starch and sucrose metabolism, two-component system, and amino sugar and nucleotide, and sugar metabolism) were enriched (*P* < 0.05) in the CON group. PCoA plots based on the Bray–Curtis distance metric revealed the differences in KEGG pathways between samples from the two dietary treatments (*P* < 0.05).

### 3.5 Co-occurrence analysis among rumen bacteria, archaea, and fungi

**FIGURE 4:**
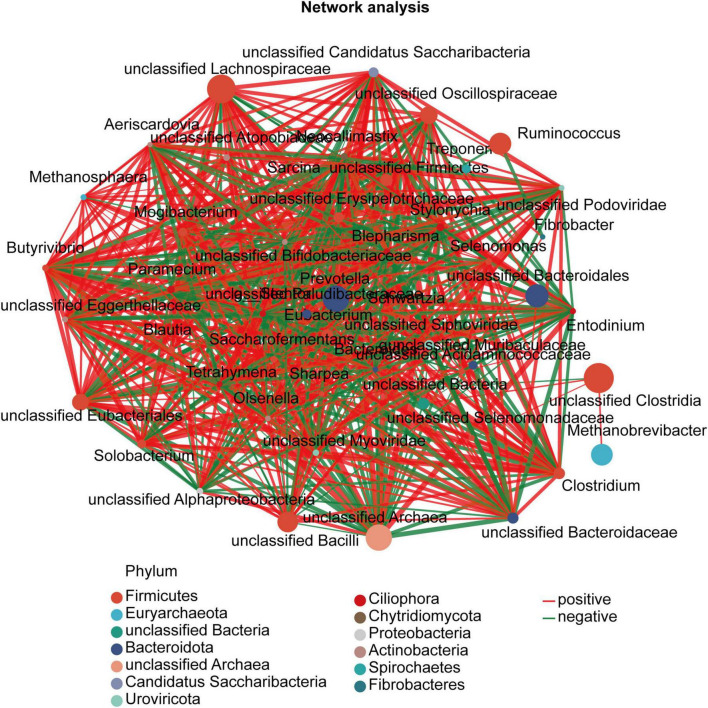
Co-occurrence network of fungal, archaeal, and bacterial genera in the rumen between different treatments. The genus correlation network maps mainly reflect the genus (relative abundance of the top 50)-relatedness under the treatment. Only significant correlations between the genera are presented (*P* < 0.05). Node size is proportional to genus abundance, while color corresponds to taxonomic classification of the phylum. The color of the lines represents a positive (red) or negative (green) correlation, and the line thickness represents the strength of the correlation.

Since rumen microbes work synergistically to perform various metabolic activities, we sought to determine the associative interactions between fungal, archaeal, and bacterial genera using co-occurrence analysis based on the top 50 genera with 47 nodes. Figure 4 presents the associations for the different treatments. To identify significant changes in the structure of the co-occurrence networks, we compared the node degree, which reflects the degree of connectivity, between the treatment groups, with only the nodes exhibiting significant correlations being presented. According to degree centrality, closeness centrality, and betweenness centrality, *unclassified Alphaproteobacteria*, *Sarcina*, *Anaeromyces*, and *unclassified Oscillospiraceae* were the core genera.

## 4 Discussion

This study was conducted to determine the impact of steam explosion and fermentation pretreatment of corn stover on the rumen microbial community and the growth performance of sheep that subsequently consumed it. A previous study demonstrated that the treatment of plant material with a combination of steam explosion and microbial degradation can overcome the drawbacks of conventional microbial pretreatment technology, and can facilitate the subsequent high-value conversion of lignocellulose (Zhang W. et al., 2023). However, the results of our study indicated that compared with the CON group, the sheep in the SEM group had lower growth performance (Table 2). Thus, our results do not support our hypothesis that the combination of steam explosion processing and fermentation treatment would promote the utilization of corn stover as sheep feed.

Many studies have demonstrated that steam explosion processing of corn stover leads to significant structural changes, such as the shortening of cellulose polymers, increased porosity of lignocellulose, and enhanced saccharification efficiency through enzymatic hydrolysis (Yang and Wang, 2019). However, in the present study (Tables 2, 3), pretreating corn stover with steam explosion and microbial degradation unexpectedly reduced the DDMI and growth performance of sheep, while also decreasing the TP and ALB concentrations in their serum. A potential explanation for these findings is that steam explosion treatment may alter the structural properties of bioactive components in plant-based materials, which may, in turn, affect their functions. Specifically, steam explosion can reduce particle size (Wang et al., 2022), increase surface area, and create larger cavities and gaps between plant cells (Cheng et al., 2020) These structural changes may influence the bioavailability and digestibility of proteins, potentially reducing their nutritional value. Moreover, excessive steam explosion reduced the biological activity of the active components in plant materials. For instance, Wenyu Cui et al. (2023) found that excessive steam explosion reduced the oil-holding capacity of grape pomace. In our study, sheep fed corn stover pretreated with steam explosion and fermentation had lower serum levels of TP and ALB, which are generally considered to be indicators of protein synthesis and metabolism in ruminants (Liu et al., 2018). These lower levels suggest that protein synthesis and absorption may have been impaired in this group (Kim et al., 2017). Thus, it is suggested that steam explosion processing would lower the nutritional value of protein in lignocellulosic biomass, consistent with the findings of a previous study on typical crop byproducts (He et al., 2022). This reduction in protein quality could be attributed to protein degradation and the Maillard reaction, both of which are induced by excessive steam explosion, leading to a decrease in crude protein content and a loss of nitrogenous nutrients (He et al., 2022). Thus, steam explosion can lower the utilization of nitrogen content by animals, which would ultimately reduce their growth performance. Before the application of steam explosion technology, more animal studies should be conducted to evaluate the effectiveness of steam-exploded corn stover on animals.

Rumen microbiota has been demonstrated to be critical to host health by providing metabolic products, maintaining metabolic function, ensuring proper development of the immune system, and defending against pathogens (Lozupone et al., 2012; Yin et al., 2023). Microbial diversity is vital for the maintenance of microbial functions in the host (Li et al., 2018); high microbial diversity stabilizes the community structure and promotes its resistance to fluctuations in the environment (Li H. et al., 2019). This explains the significant associations of rumen microorganisms with host performance in the current study. This is supported by previous studies showing a difference in microbial diversity between steers with efficient or inefficient use of feed (higher alpha diversity was observed in the efficient group; (Li F. et al., 2019), and showing that a more diverse rumen microbial community can promote the use of high-fiber herbage in yaks (Fan et al., 2020). Research has also revealed that the diversity of human intestinal microbiota improves the efficiency of dietary fiber fermentation (Tap et al., 2015), and reflects better health and strong metabolic capacity (Clarke et al., 2014). Meanwhile, in our study, the rumen microbial diversity in the SEC group was lower than that in the CON group. This may be explained by various toxins being generated during steam explosion processing, which may impede microbial growth or enzymatic hydrolysis (Nie et al., 2021). Thus, we speculate that a failure to accurately control the strength and uniformity of steam explosion treatment may produce compounds that adversely affect microbiota composition, abundance, and reproduction.

Diet is considered to be the most important factor determining microbial composition (O’Hara et al., 2020). The microbiome plays a critical role in providing nutrition to host ruminants, thereby influencing their performance (Huang et al., 2021). Previous research has shown that the genus *Eubacterium*, as symbiotic bacteria, may play a vital role in feed digestion (Mao et al., 2012) and residual feed intake (Hernandez-Sanabria et al., 2012) in cattle, with it being able to regulate the micro-ecological balance by producing short-chain fatty acids (Dadi et al., 2020). Meanwhile, both *Eubacterium* and *Methanosphaera* have been negatively associated with methane production by depriving methanogens of hydrogen for methanogenesis, resulting in less CH_4_ production per unit of carbon (Cunha et al., 2019). In the present study (Figure 2A), the genera *Eubacterium* and *Methanosphaera* were distinct signature taxa in the rumen of the CON group, suggesting that they contribute to CH_4_ reduction, thereby reducing the loss of energy in the activity of the microbial community, and in turn, improving the utilization of feed. Meanwhile, the genus *Clostridium*, which is associated with cellulose degradation (Huws et al., 2018) and nitrogen fixation (Noguchi et al., 2006), was one of the distinct signature taxa in the rumen of the CON group. In contrast, several genera belonging to the phylum Ciliophora (*Ichthyphthirius*, *Stentor*, *Stylonychia*, *Tetrahymena*, *Pseudocohnilembus*, and *Paramecium*) and the genus *Neocallimastix*, which showed positive correlations with methane were significantly more abundant in the SEC group (Saborío-Montero et al., 2020). Moreover, a previous study demonstrated that, in cows with inefficient feed utilization, rumen metabolism shifted toward waste products such as methane, as evidenced by the specific enrichment of methane-related species and the increase in methane production (Shabat et al., 2016). Ruminants emit enteric CH_4_ as a byproduct of feed degradation and fermentation processes, which is mainly exhaled/eructated and not retained by the host. The loss of energy associated with the production of methane, which is estimated to vary between 2 and 12% of gross energy consumption, is metabolically undesirable as this energy could otherwise be used in beneficial ways (Saborío-Montero et al., 2020). An *in vitro* study also demonstrated that, because the improvement of fermentation efficiency produced more fermentation products, more methane was produced to remove metabolic hydrogen and keep the H_2_ partial pressure low (Wang et al., 2020). Thus, we speculated that the application of steam explosion and fermentation pretreatment may make corn stover more easily degradable, but it may also change the ruminal microbial community, lower the population and reproduction of several microbial species, and increase methane-related species, thus reducing the efficiency of feed utilization. Our results suggest that the effects of this pretreatment combination on the nutritional composition, functional components, and physicochemical properties of plant-based raw materials for ruminant consumption need to be further investigated before it can be widely applied. To address the negative effects observed in the SEC group, further research should explore potential strategies to mitigate these effects. For instance, adjusting the fermentation process by increasing the pressure, as demonstrated by Kong et al. (2022) and Zhang W. et al. (2023), may be a beneficial strategy to improve the utilization efficiency of the feed (Kong et al., 2022). Additionally, optimizing the proportion of steam-exploded corn stover in the diet, combined with the use of alternative microbiota, such as cellulase and lactic acid bacteria, has been shown to enhance the degradation of lignocellulosic material and improve fermentation efficiency (Nie et al., 2021; Zhang H. et al., 2023) could be considered potential solutions to enhance the utilization efficiency of the feed. These strategies may help improve microbial activity and fermentation efficiency, ultimately leading to better feed conversion and growth performance in ruminants. Future studies will focus on evaluating these approaches to determine their effectiveness in improving the nutritional value of steam-exploded corn stover and mitigating the observed negative effects.

In addition, the results of the KEGG pathway analysis indicated that the different types of corn stover significantly impacted the gene functional profiles in the sheep rumen microbiome (Figure 3). Notably, in the rumen microbiome of sheep fed the untreated corn stover, ABC transporters were the most expressed pathway in membrane transport, which directly participate in ATP production. These transporters also act as a barrier to protect ruminants from the invasion of toxic substances into the stomach (Hamana et al., 2012). The lower expression of ABC transporters in the SEC group indicates that less ATP may be produced in this group. The decrease in the relative abundance of the major microbial phyla and the lower Chao1 index (indicating lower microbial diversity) in the SEC group could explain the decrease in microbial activities in the rumen. Although we identified several specific taxa and KEGG pathways associated with the consumption of corn stover after its processing by steam explosion and fermentation in combination, the mechanisms behind these changes and their impact on rumen activity remain unclear (Figure 4). Although our findings provide important insights, the application of steam explosion and fermentation pretreatment to corn stover requires the need for further study such as, for example, a focus on animal utilization, before this approach can be widely applied.

## 5 Conclusion

Our results demonstrated that feeding sheep corn stover pretreated by steam explosion and fermentation can disrupt the balance of their rumen microbiota. The diversity and relative abundance of taxa associated with lower methane production were significantly reduced in the SEC group. We hypothesized that, compared to the CON group, sheep in the SEC group may have experienced greater energy loss due to increased methane production and less active microbial populations, leading to inefficient feed utilization. Methane production represents a loss of energy that could otherwise be used for growth and metabolic processes. Furthermore, the reduced microbial diversity observed in the SEC group likely contributed to decreased fermentation efficiency, further reducing energy availability. These factors could explain the reduced feed intake and lower microbial diversity, ultimately resulting in poorer growth performance in the SEC group. However, these hypotheses are speculative and require further validation. Future studies should focus on evaluating the digestibility and bioavailability of both untreated and steam-exploded corn stover to gain a deeper understanding of its nutritional impact. Additionally, exploring rumen fermentation parameters will offer valuable insight into the broader implications of steam explosion processing, especially in terms of energy loss and nitrogen utilization in ruminants. In addition, further optimization of the production process, with particular attention paid to the proportion of corn stover in the diet, is needed before the widespread application of steam explosion to corn stover.

## Data Availability

The datasets presented in this study can be found in online repositories. The names of the repository/repositories and accession number(s) can be found in the article/supplementary material.

## Footnotes

1
www.majorbio.com

2
https://github.com/OpenGene/fastp

3
https://github.com/voutcn/megahit

4
http://metagene.cb.k.u-tokyo.ac.jp/

5
http://www.ncbi.nlm.nih.gov/Taxonomy/taxonomyhome.html/index.cgi?chapter=tgencodes#SG1

6
https://github.com/drostlab/rdiamond

7
https://www.genome.jp/kegg/
